# Grinding Apparatus

**Published:** 1850-01

**Authors:** John Harris

**Affiliations:** Salem, O.


### GRINDING APPARATUS.

Messrs. Editors.—Having been in the habit of using a grinding apparatus for two years past, constructed on a plan somewhat different from that in common use, I thought it might not be uninteresting to your readers to have a description of it.

It consists of a piece of wood three inches by four in thickness, ten inches long, with two upright pieces of wood, firmly morticed into the first piece at right angles, and three inches apart. Three iron shafts pass between these upright pieces, the upper one long enough to reach two inches beyond one of the uprights; upon the end of this shaft the emery wheels, brush, &c., are fastened by means of a bur. The middle shaft, by means of a handle, turns a cog wheel ten inches in diameter, which connects with one on the lower shaft, of one and a half inch diameter. The upper and lower shafts are connected by a strap and pullies. The object of the cog wheels and pullies is to multiply the revolutions of the emery wheel, which it does twenty times. The fixture can be screwed upon a bench, or what is preferable, it can be framed into a bench, so as to bring the emery wheels, brushes, &c., to any desirable distance above the bench.

Amongst the advantages which I suppose it to possess over other fixtures which I have seen, are first, the complete control of the velocity of the wheels, &c.; second, the small size and portability of the apparatus; and third, its cheapness—mine not costing four dollars.

Yours, &c., JOHN HARRIS.

P. S.—Since writing the above, I have been using one of Dr. Van Emen's bellows, which I had constructed five or six weeks ago. I find it to be a most efficient little apparatus, fulfilling the purposes for which it is designed, in a most admirable manner.

I notice in the Dental News Letter, a description of a bellows somewhat similar to the above, to be compressed between the knees, or by a treddle. It strikes me that the one invented by Van Emen is the preferable apparatus. J. H.

SALEM, O., Nov. 9th, 1849.

